# Suicide Attempts in Michigan HealthCare System; Racial Differences

**DOI:** 10.3390/brainsci8070124

**Published:** 2018-06-30

**Authors:** Shervin Assari

**Affiliations:** 1 Department of Psychiatry, University of Michigan, 4250 Plymouth Rd., Ann Arbor, MI 48109-2700, USA; assari@umich.edu; Tel.: +1-734-232-0445; 2 Center for Research on Ethnicity, Culture, and Health (CRECH), University of Michigan School of Public Health, Ann Arbor, MI 48109-2029, USA; 3 Department of Psychology, University of California Los Angeles (UCLA), Franz Hall, 502 Portola Plaza, Los Angeles, CA 90095, USA; 4 BRITE Center for Science, Research and Policy, University of California, Los Angeles (UCLA), Los Angeles, CA 90095, USA

**Keywords:** ethnic groups, blacks, whites, suicide attempt, suicide, depression, insurance, comorbidity, physical health

## Abstract

Background. Community-based studies have documented racial differences in social, psychiatric, and medical determinants of suicidal ideation; however, less is known about racial differences in the profile of suicide attempts in clinical settings. Aim. The current study aimed to compare Blacks and Whites who attempted suicide for demographic factors, socioeconomic status, medical history, psychiatric disorders, and outcomes. Methods. This retrospective study was a retrospective chart review of DataDirect, which is an electronic data repository of the Michigan Health Care System, 2014 to 2017. This analysis included 6147 suicide attempts (5388 Whites and 759 Blacks). Race, sociodemographic factors, medical history, psychiatric disorders, and outcomes were measured. Results. Blacks and Whites with suicide attempt did not differ in age or gender, but varied by insurance type. Blacks were more commonly under Medicare and Medicaid, while Whites were more commonly under private insurance or self-pay (*p* < 0.05). Blacks with suicide attempt were more likely to be obese, while Whites with suicide attempt were more likely to be underweight. Frequency of psychiatric disorders, including depression, alcohol abuse, drug abuse, and psychosis, were not different between Whites and Blacks with suicide attempt; however, medical conditions showed a different profile across racial groups. When compared to Whites, Blacks had higher prevalence of uncomplicated hypertension, renal failure, chronic obstructive pulmonary disease, coagulopathy, and obesity (*p* < 0.05 for all comparisons). In contrast, Whites had higher prevalence of other neurological disorders than Blacks. There were no differences in in-patient survival rate between Whites and Blacks who attempted suicide. Conclusion. There are considerable differences between Blacks and Whites with at least one suicide attempt. Although their psychiatric diagnoses seem to be similar, Blacks who have attempted suicide attempt have more medical comorbidities than their White counterparts. Lack of racial disparities in in-patient mortality rate of suicide attempts in the Michigan Health Care System is promising news given the higher physical health needs of Blacks when compared to Whites.

## 1. Introduction

A wide range of social [1,2,3,4], medical [5], and psychiatric disorders [6] increase the risk of suicide. Various forms of low socioeconomic status, such as poverty, unemployment, low education, and being single, increase suicide risk [2]. A wide range of psychiatric disorders, such as depression, anxiety, and substance use disorder, are among the most well-established risk factors of suicide [7,8,9,10], with number of psychiatric disorders being one of the strongest predictors of suicidal behaviors [11]. A considerable proportion of individuals with a suicide attempt have at least one psychiatric disorder, with depression being the most common [5]. Psychosocial and cognitive constructs, such as hopelessness, are also among independent and fundamental risk factor for suicidal behaviors [12,13,14,15,16,17]. Health problems that have low survival rate, such as cancer and cardiovascular disease, are also among the major risk factors for suicide, particularly among older men [18].

However, there is considerable evidence originating from community samples that suggests, similar to other health outcomes [19,20], that the risk factors of suicide may depend on race and ethnicity [19,21,22]. For example, Whites and Blacks differ in the additive and multiplicative effects of socioeconomic status, religiosity, and psychiatric disorders on suicide [19,23]. Multiple psychiatric disorders have sub-additive effects among Whites [24,25,26,27], but these effects seem to be synergistic for Blacks [23]. Even within Blacks, major ethnic differences exist in the types of psychiatric disorders that are relevant to suicidality [21,28]. In the general population of adults, general anxiety disorder seems to be a more salient risk factor for suicidal ideation in African Americans and drug abuse seems to be a more salient risk factor for Caribbean Blacks [28]. Some research also suggests Whites and Blacks differ in the mental health effects of socioeconomic status, such as education and income [21]. In a study, high education was positively associated with suicidal ideation among Black women, a pattern that was not found in Black men or White men or women [21].

Suicide has been historically seen as a public health problem for Whites, but not Blacks [29,30]. However, the suicide rate among Blacks has recently increased [31,32]. There are even reports suggesting that Blacks and Whites experience similar rates of suicide attempt [33]. Still, limited information exists on how risk factors of suicide differ between Blacks and Whites [19,21,23,28].

Although previous research has compared Blacks and Whites for the effects of social and psychiatric conditions on suicidal ideation, there is still a need for additional research on racial differences in risk factors of suicide attempt for at least four reasons. First, most of the existing knowledge on Black-White differences in predictors of suicide is limited to suicidal ideation, leaving a knowledge gap in racial variation of suicide attempts. Second, most health disparity literature on racial differences in determinants of suicide is from national epidemiological studies of community samples. Thus, less is known about the same racial differences in clinical settings. Third, there are many instances where the results of community samples are not replicated in a clinical setting. Finally, there is not much known about Black-White differences in medical history and mortality rates of patients who attempt suicide and receive care.

In continuation of our previous research on the heterogeneity of the risk factors of suicidal ideation based on race and ethnicity [19,21,23,34], we used data from DataDirect, which is an electronic data repository from the University of Michigan Health System (UMHS), to compare sociodemographic, medical, psychiatric, and outcome data of suicide attempts between Whites and Blacks. We hypothesize that socioeconomic status (SES) [21], which is a major depressive disorder [35,36,37,38], and drug use [28] may play different roles in suicide attempts of Whites and Blacks. We also hypothesize higher mortality rate of admissions due to suicide attempt in Blacks when compared to Whites.

## 2. Methods

Design and Settings. This retrospective chart review study used data from DataDirect, University of Michigan (https://datadirect.med.umich.edu/). DataDirect is a self-service website enabling access to clinical data such as diagnoses, encounters, procedures, medications (ordered and administered), and labs on more than four million unique patients from across the UMHS enterprise. DataDirect provides aggregate counts for cohort discovery and the ability to download patient health data.

Ethics. The current study used only aggregates and not patient level data. Since individual level characteristics were not used and the data set did not have any identifier, the study was exempt from institutional review board (IRB) review.

Participants. To recruit patients in this study, a census approach was used. All of the patients who used services for suicidal attempt(s) were included in this study.

Demographic characteristics. Demographic characteristics included gender (male and female) and age (18–30 years, 31–40 years, 41–50 years, 51–60 years, 61–70 years, 71–80 years, and other).

Medical Insurance. Medical Insurance included private insurance, self-pay, Medicaid, Medicare, Other Governmental Insurance, Workers Compensation, Other, and Unknown.

Body Mass Index (BMI). BMI was objectively measured. In line with the WHO definitions, BMI categories were below 18.5, 18.5–24.9, 25.0–29.9, 30.0–34.9, 35.0–39.9, and above 40. These categories reflect underweight, normal weight, overweight, obese class I, obese class II, and obese class III, respectively [39,40].

Psychiatric comorbidities. Psychiatric conditions included depression, alcohol abuse, drug abuse, and psychoses. These diagnoses were based on ICD-9 and ICD-10 codes.

Medical comorbidities. Comorbid conditions included congestive heart failure, valvular disease, peripheral vascular disorders, hypertension complicated, hypertension uncomplicated, peptic ulcer disease, excluding bleeding, renal failure, rheumatoid arthritis collagen vascular diseases, chronic pulmonary disease, liver disease, pulmonary circulation disorders, blood loss anemia, cardiac arrhythmias, coagulopathy, deficiency anemia, diabetes complicated, diabetes uncomplicated, obesity, hypothyroidism, fluid electrolyte disorders, lymphoma, metastatic cancer, solid tumor without metastasis, weight loss, other neurological disorders, and paralysis.

Suicidal attempt. Suicidal attempt was defined based on ICD-9 and ICD-10 codes. The ICD-9 codes included is listed in the Appendix A.

### Statistical Analysis

We used SPSS 21.0 (SPSS Inc., Chicago, IL, USA) for data analysis. Data analysis included univariate and bivariate analyses. For univariate analysis, we reported frequencies and relative frequencies (proportions) in the pooled sample and by race. For bivariate analyses, we used the Pearson Chi square to test the association between race and our social, clinical, and outcome variables. P less than 0.05 was considered to be significant.

## 3. Results

### 3.1. Descriptive Statistics

Table 1 presents descriptive statistics regarding age, gender, and insurance type in the overall sample, as well as Blacks and Whites who were admitted for suicidal attempt. About 62% of the patients were female, but there was no significant difference in gender of Blacks and Whites who were admitted (*p* > 0.05). Blacks and Whites were also not different by age distribution (*p* > 0.05). However, Blacks and Whites showed a significant difference in their insurance type. Blacks were more commonly under Medicare and Medicaid, while Whites were more commonly insured as private insurance or self-pay (*p* < 0.05 for all comparisons). (Table 1)

### 3.2. Body Mass Index

Whites who attempted suicide were more commonly in normal weight when compared to Blacks. In addition, Blacks were more commonly in obese class I, obese class II, and obsess class III categories as compared to Whites (*p* < 0.05 for all comparisons). (Table 2)

### 3.3. Psychiatric Diagnoses

Blacks and Whites did not significantly differ in frequency of psychiatric conditions, including depression, alcohol abuse, drug abuse, and psychoses (*p* > 0.05). (Table 3)

### 3.4. Medical Diagnoses

When compared to Whites, Blacks had a higher prevalence of hypertension uncomplicated, renal failure, chronic obstructive pulmonary disease, coagulopathy, and obesity (*p* < 0.05 for all comparisons). In contrast, Whites had a higher prevalence of other neurological disorders than Blacks (*p* < 0.05). (Table 4)

### 3.5. Inpatient Mortality

From all the patients examined, 245 died (4.40%), while 5323 patients survived (95.60%). From the patients who died (*n* = 245), 24 were Blacks (9.80%) and 221 were Whites (90.20%). Death rate was 3.16% (24 out of 759 total) in Blacks and 4.10% (221 out of 5388 total) in Whites. There were no differences in the survival rate of Whites and Blacks.

## 4. Discussion

Comparison of suicide attempts in a large healthcare system showed similarities and differences between Blacks and Whites. Age, gender, and psychiatric profile of patients did not differ by race. Blacks and Whites, however, differed in insurance type and physical health. Unexpectedly, there were no racial disparities in inpatient mortality rate of patients with suicide attempt. Given that Blacks had worse physical health and the fact that health disparities are rules rather than exceptions, no disparities in inpatient mortality rate of Blacks’ and Whites’ suicide attempts seem to be very promising.

The result of this study extends the current knowledge on suicide attempts from a community to clinical setting. Epidemiological and survey results have shown that racial and ethnic differences exist in contribution of SES and psychiatric conditions on suicidal ideation. For instance, Whites have sub-additive effects of multiple psychiatric behaviors [24,25,26], while these effects are multiplicative for Blacks [22,23]. Although community-based studies have shown that multiple psychiatric conditions, such as depression, drug abuse, alcohol use, and anxiety, interact differently on suicidal ideation, Blacks and Whites with a history of attempted suicide did not differ in their psychiatric diagnoses [22,23,24,25,26]. 

Previous research by Joe et al. has shown that the risk of suicide attempt in Blacks may increase up to eight times in the presence of psychiatric disorders when compared to those without a psychiatric disorder [41]. Similarly, strong association also exists in Blacks between psychiatric disorders and suicidal ideation [41]. Assari et al. documented a dose-dependent effect of number of psychiatric disorders on age of first serious suicidal ideation among Blacks. That is, Blacks with two or more psychiatric disorders had an earlier age of onset of suicidal ideation when compared to those with one psychiatric disorder, and those who have one psychiatric disorder had started their suicidal ideation earlier than those who do not have any psychiatric disorders [42]. In a recent study, number of lifetime psychiatric disorders was a risk factor for lifetime suicidal ideation for all ethnic by gender groups of Blacks [43].

Screening for suicide and emotional problems, particularly depression, needs to consider the racial variations in experience and expression of negative emotions and cognitions [44,45]. Multiple studies have shown differences between Blacks and Whites in how poor self-evaluation of mental health reflects psychiatric disorders, such as depression [46,47]. Overall, depression better translates in perceived and reported mental health problems for Whites than Blacks [46,47]. Since Blacks tend to underreport their own needs, Blacks and Whites who report similar self-rated mental health may require different health needs [46]. There may also be risk factors specific to racial groups [3]. A recent review discussed the differences between Blacks and Whites in social, emotional, and cognitive correlates of depression [48]. Some studies showed Blacks who are depressed maintain higher levels of overall sense of mastery and hope, which may reduce their suicidality [37,38,49,50,51]. However, higher education may not be as protective for Blacks as it is for Whites [21]. Another major difference is chronic medical conditions, which were more closely associated with depression among Blacks at each time point [52]; however, physical health and depression may be better linked over time for Whites than Blacks [53,54,55,56]. Many of these racial differences have implications for mental health screening and suicide prevention of individuals based on race. Future research should test how tailoring an intervention enhances the performance and utility of screening and diagnostic protocols. 

Our findings highlight both the universal and race-specific strategies that may be needed to treat suicide attempts of Whites and Blacks. Universal programs are needed for psychiatric conditions; however, more medical interventions are needed for Blacks who attempt suicide. This finding should not undermine the importance of suicide prevention of individuals with risk factors, such as psychiatric disorders.

Psychiatric disorders are the strongest risk factor for suicide, at least in industrial countries [11], and having multiple psychiatric conditions disproportionately increases the risk of suicidality [23,42]. However, our findings showed a considerable proportion of individuals with a suicide attempt did not have a comorbid psychiatric condition, regardless of race. Thus, clinicians, public health experts, and policy makers should not assume that suicide attempts only occur among those who have a diagnosable psychiatric disorder. This finding suggests that the evaluation of and screening for suicide should not be limited to individuals with serious mental health problems (i.e., psychiatric disorders).

## 5. Limitations

The current study had a few limitations. First, this was a retrospective electronic chart review. As a result, causative conclusions are not plausible. The sample size was imbalanced, with a disproportionately smaller sample size of Blacks with a suicidal attempt. This may have implications for statistical power of this study. Furthermore, the sample of this study was not representative of the United States (U.S.), so the results should not be generalized to the racial landscape of suicide in the U.S. In addition, our list of variables was not a comprehensive list [4,57,58,59,60,61,62,63,64,65,66,67,68]. Several relevant constructs, including other risk factors of suicide, were not evaluated. Finally, this study did not study the intersection of race and gender. Gender may alter correlates of suicide in Whites and Blacks.

This was a chart review of administrative but not a research dataset. Although this increases the external validity of the study, the current study has low internal validity because it was not a research study with specific measures, protocol, or eligibility. Future research should replicate these findings with adjustment for multiple comparisons. This is particularly important given the simple analysis that was used for this study. Additionally, future research should go beyond a comparison of Blacks and Whites, as well as control for confounding factors and test for moderation and mediations. A multivariate analysis was not possible in this study because we did not have access to individual level data (This analysis only used aggregate data).

## 6. Conclusions

Both similarities and differences exist in the profile of Blacks and Whites with a suicide attempt in a tertiary health care system in the Midwest. As a result, both universal and racially tailored programs are needed for treatment and prevention of suicide attempts. Blacks who need care for suicide need more comprehensive health care that covers their physical health needs.

## Figures and Tables

**Table 1:** Demographic and insurance characteristics for Blacks (*n* = 759) and Whites (*n* = 5388) with suicidal attempt.

	Blacks		Whites		All	
	*n*	%	*n*	%	*n*	%
Gender						
Male	297	39.13	2046	37.97	2343	38.12
Female	462	60.87	3342	62.03	3804	61.88
Age						
18–30	270	35.57	2002	37.16	2272	36.96
31–40	90	11.86	671	12.45	761	12.38
41–50	106	13.97	661	12.27	767	12.48
51–60	65	8.56	486	9.02	551	8.96
61–70	27	3.56	133	2.47	160	2.60
71–80	0	0.00	33	0.61	35	0.57
Other	194	25.56	1402	26.02	1596	26.05
Insurance						
Private Insurance *	260	34.26	3233	60.00	3493	56.82
Self-Pay *	45	5.93	454	8.43	499	8.12
Medicaid *	346	45.59	1373	25.48	1719	27.96
Medicare *	115	15.15	524	9.73	639	10.40
Other Governmental Insurance *	47	6.19	206	3.82	253	4.12
Workers Compensation	0	0.00	15	0.28	15	0.24
Other	<10	-	106	1.97	115	1.87
Unknown	25	3.29	82	1.52	107	1.74

* *p* < 0.05.

**Table 2:** Body mass index characteristics for Blacks (*n* = 759) and Whites (*n* = 5388) with suicidal attempt.

	Blacks		Whites		All	
	*n*	%	*n*	%	*n*	%
Below 18.5	31	4.08	238	4.42	269	4.38
18.5–24.9 *	170	22.40	1731	32.13	1901	30.93
25.0–29.9	133	17.52	896	16.63	1029	16.74
30.0–34.9 *	109	14.36	428	7.94	537	8.74
35.0–39.9 *	62	8.17	205	3.80	267	4.34
Above 40 *	49	6.46	223	4.14	272	4.42
Unknown *	205	27.01	1667	30.94	1872	30.45

* *p* < 0.05.

**Table 3:** Psychiatric disorders among Blacks (*n* = 759) and Whites (*n* = 5388) with suicidal attempt.

	Blacks		Whites		All	
	*n*	%	*n*	%	*n*	%
Depression	186	24.51	1252	23.24	1458	23.72
Alcohol Abuse	<10	-	21	0.39	24	0.39
Drug Abuse	61	8.04	384	7.13	447	7.27
Psychosis	27	3.56	137	2.54	164	2.67

**Table 4:** Medical comorbidities in Blacks (*n* = 759) and Whites (*n* = 5388) with suicidal attempt.

	Blacks		Whites		All	
	*n*	%	*n*	%	*n*	%
Congestive Heart Failure	<10	-	30	0.56	34	0.55
Valvular Disease	0	0.00	14	0.26	14	0.23
Peripheral Vascular Disorders	<10	-	13	0.24	14	0.23
Hypertension Complicated	<10	-	17	0.32	22	0.36
Hypertension Uncomplicated *	46	6.06	161	2.99	208	3.38
Peptic Ulcer Disease Excluding Bleeding	0	0.00	10	0.19	10	0.16
Renal Failure *	10	1.32	30	0.56	40	0.65
Rheumatoid Arthritis Collagen Vascular Diseases	<10	-	26	0.48	30	0.49
Chronic Pulmonary Disease *	49	6.46	208	3.86	257	4.18
Liver Disease	<10	-	73	1.35	76	1.24
Pulmonary Circulation Disorders	<10	-	20	0.37	26	0.42
Blood Loss Anemia	0	0.00	23	0.43	23	0.37
Cardiac Arrhythmias	24	3.16	211	3.92	235	3.82
Coagulopathy *	20	2.64	46	0.85	66	1.07
Deficiency Anemia	<10	-	19	0.35	22	0.36
Diabetes Complicated	<10	-	13	0.24	18	0.29
Diabetes Uncomplicated	13	1.71	82	1.52	95	1.55
Obesity *	38	5.01	112	2.08	150	2.44
Hypothyroidism	<10	-	64	1.19	67	1.09
Fluid Electrolyte Disorders	15	1.98	88	1.63	103	1.68
Lymphoma	0	0.00	< 10	-	< 10	-
Metastatic Cancer	0	0.00	18	0.33	18	0.29
Solid Tumor Without Metastasis	<10	-	20	0.37	22	0.36
Weight Loss	<10	-	49	0.91	51	0.83
Other Neurological Disorders *	12	1.58	173	3.21	185	3.01
Paralysis	<10	-	< 10	-	10	0.16

* *p* < 0.05.

## Appendix A.

### The ICD-9 Codes Used to Define Suicidal Attempt

The ICD-9 codes:

Suicide and self-inflicted injury by unspecified means (E958.9), suicide and self-inflicted injury by jumping or lying before moving object (E958.0), suicide and self-inflicted injury by burns, fire (E958.1), suicide and self-inflicted injury by scald (E958.2), suicide and self-inflicted injury by extremes of cold (E958.3), suicide and self-inflicted injury by electrocution (E958.4), suicide and self-inflicted injury by crashing of motor vehicle (E958.5), suicide and self-inflicted injury by crashing of aircraft (E958.6), suicide and self-inflicted injury by caustic substances, except poisoning (E958.7), suicide and self-inflicted injury by other specified means (E958.8), suicide and self-inflicted poisoning by analgesics, antipyretics, and antirheumatics (E950.0), suicide and self-inflicted poisoning by barbiturates (E950.1), suicide and self-inflicted poisoning by other sedatives and hypnotics (E950.2), suicide and self-inflicted poisoning by tranquilizers and other psychotropic agents (E950.3), suicide and self-inflicted poisoning by other specified drugs and medicinal substances (E950.4), suicide and self-inflicted poisoning by unspecified drug or medicinal substance (E950.5), suicide and self-inflicted poisoning by agricultural and horticultural chemical and pharmaceutical preparations other than plant foods and fertilizers (E950.6), suicide and self-inflicted poisoning by corrosive and caustic substances (E950.7), and suicide and self-inflicted poisoning by arsenic and its compounds (E950.8), suicide and self-inflicted poisoning by other and unspecified solid and liquid substances (E950.9).

The ICD-10 codes:

Suicide attempt <T14.91> that included intentional self-harm by other specified means, initial encounter <X83.8XXA>, intentional self-harm by jumping or lying in front of motor vehicle, subsequent encounter <X81.0XXD>, intentional self-harm by jumping or lying in front of other moving object, subsequent encounter <X81.8XXD>, Intentional self-harm by jumping or lying in front of motor vehicle, initial encounter <X81.0XXA>, intentional self-harm by jumping or lying in front of other moving object, initial encounter <X81.8XXA>, intentional self-harm by jumping or lying in front of (subway) train, initial encounter <X81.1XXA>, intentional self-harm by other hot objects, initial encounter <X77.8XXA>, intentional self-harm by smoke, fire and flames, initial encounter <X76.XXXA>, intentional self-harm by smoke, fire and flames, subsequent encounter <X76.XXXD>, intentional self-harm by unspecified hot objects, initial encounter <X77.9XXA>, intentional self-harm by hot household appliances, initial encounter <X77.3XXA>, intentional self-harm by unspecified hot objects, subsequent encounter <X77.9XXD>, intentional self-harm by hot household appliances, subsequent encounter <X77.3XXD>, intentional self-harm by steam or hot vapors, initial encounter <X77.0XXA>, intentional self-harm by hot tap water, initial encounter <X77.1XXA>, intentional self-harm by other hot fluids, subsequent encounter <X77.2XXD>, intentional self-harm by other hot fluids, initial encounter <X77.2XXA>, intentional self-harm by exposure to extremes of cold, initial encounter <X83.2XXA>, intentional self-harm by exposure to extremes of cold, subsequent encounter <X83.2XXD>, intentional self-harm by electrocution, initial encounter <X83.1XXA>, Intentional self-harm by electrocution, subsequent encounter <X83.1XXD>, intentional collision of motor vehicle with other motor vehicle, subsequent encounter <X82.0XXD>, intentional collision of motor vehicle with tree, initial encounter <X82.2XXA>, other intentional self-harm by crashing of motor vehicle, subsequent encounter <X82.8XXD>, intentional collision of motor vehicle with other motor vehicle, initial encounter <X82.0XXA>, intentional collision of motor vehicle with train, subsequent encounter <X82.1XXD>, intentional collision of motor vehicle with train, initial encounter <X82.1XXA>, other intentional self-harm by crashing of motor vehicle, initial encounter <X82.8XXA>, intentional self-harm by crashing of aircraft, subsequent encounter <X83.0XXD>, intentional self-harm by crashing of aircraft, initial encounter <X83.0XXA>, intentional self-harm by blunt object, subsequent encounter <X79.XXXD>, intentional self-harm by other specified means, subsequent encounter <X83.8XXD>, intentional self-harm by blunt object, initial encounter <X79.XXXA>, poisoning by unspecified antiepileptic and sedative-hypnotic drugs, intentional self-harm, initial encounter <T42.72XA>, poisoning by unspecified drugs, medicaments and biological substances, intentional self-harm, initial encounter <T50.902A>, poisoning by other drugs, medicaments and biological substances, intentional self-harm, initial encounter <T50.992A>, toxic effect of unspecified corrosive substance, intentional self-harm, initial encounter <T54.92XA>, toxic effect of arsenic and its compounds, intentional self-harm, initial encounter <T57.0X2A>, and toxic effect of unspecified substance, intentional self-harm, initial encounter <T65.92XA>.
